# Rationale for the Use of Prostaglandins and Phosphodiesterase Inhibitors in the Treatment of Functional Bladder Disorders

**DOI:** 10.5812/numonthly.14088

**Published:** 2013-11-13

**Authors:** Mohammad Sajjad Rahnama'i, Gommert A. Van Koeveringe, Philip E. Van Kerrebroeck

**Affiliations:** 1 Maastricht University Medical Centre (MUMC+), Maastricht, The Netherlands

**Keywords:** Urinary Bladder, Phosphodiesterase Inhibitors, Prostaglandin, Receptors, Prostaglandin E, EP1 Subtype, Receptors, Prostaglandin E, EP2 Subtype

## Abstract

In this paper a general discussion of the available data on the role of prostaglandin (PG) and phosphodiesterase is discussed. Functional studies would be a next step to understand the functional meaning of the data described in this paper. The data presented are a basis for further research on selective modulation of the EP1 and EP2 receptor which could be a therapeutic target in functional bladder disorders such as OAB. PDE inhibitors are closer to clinical use, as these drugs have been studied and registered for other indications such as erectile dysfunction in men. Therefore, in vivo studies in human subjects can be conducted on short term. However, from a scientific point of view, it is very important to unravel the exact site of action and role of PDE inhibition with in vitro and in vivo studies as is the case with PG. In this way, a combination of drugs targeting different mechanisms involved in bladder physiology such as PG, cGMP, cAMP, and muscarinic receptors, could reduce side effects and improve efficacy.

## 1. Introduction

The lack of satisfactory therapeutic options for the symptoms of the overactive bladder syndrome (OAB) is mainly due to an incomplete understanding of the complex bladder physiology and the multifactorial cause of the OAB symptoms. In this paper, it is aimed to address two local control systems of the bladder; namely: the prostaglandin E2 (PGE_2_) and the phosphodiesterase (PDE) - NO-cGMP system. Signal transduction is one of the most fundamental processes underlying the basics of living organisms. This process includes the recognition of signals by cells and their appropriate transformation into biological responses (1). It has become evident that neurons can also communicate with their targets without synaptic connections and that this form of non-synaptic interactions is of physiological significance (2). Therefore, a disruption of this mechanism can be of pathological significance (2). In the bladder, a part of such non synaptical cellular signal is mediated through prostaglandin and the NO/cGMP pathway.

Both clinical and basic researchers try to gain a better understanding of the principals involved in the integrated control of the lower urinary tract. To this end, the scientifically preferable study subjects are human subjects. However, legal, ethical and moral issues make experiments in human subjects difficult and in some cases even impossible. Moreover, it is often not possible to acquire enough human bladder tissue to conduct all the in vitro experiments needed. Therefore, the use of animal models in this field of research is necessary. Much of bladder research has been done in the guinea pig bladder as it shows significant structural similarities with the human bladder (3-6). Previous studies have shown that, obstructed guinea pig bladders show similar cystometric changes as in patients suffering from OAB (3-6).

## 2. EP1 and EP2 Distribution in the Bladder Wall

From all prostanoids, PGE_2_ has been put forward as the most likely candidate to contribute to overactivity of the urinary bladder (7-9). This is due to the presence of clear evidence that PGE_2_ infused into the bladder reduces bladder capacity (10-13). Furthermore, it has been shown that in detrusor overactivity models (14, 15), as well as in patients with symptoms of overactive bladder syndrome (16-18) local PGE_2_ production in the bladder is increased. PGE_2_ is a subtype of prostaglandin (PG) targeting the EP receptors that mediate its physiological effect (19). There are four subtypes (EP1-EP4), each responding to the natural agonist PGE_2_ in a different manner (20).

Each of the EP receptors uses a different G-protein coupled transduction system (21, 22). EP1 and EP3 are thought to cause contraction of the smooth muscle, whereas EP2 and EP4 are thought to cause relaxation (21, 22). This difference in response of the muscle to each of the EP receptors has been shown in the uterine smooth muscle (23). EP1 receptors are found in many tissues in which intracellular signals are generated in response to PG, involving diacylglycerol and 1,4,5-triphosphate (24). In such systems, EP1 is involved in regulating intracellular Ca^2+^ and cell excitability. EP2 receptors are coupled to a G-protein and their stimulation results in increased formation of cAMP. This rise in cAMP through EP2 then leads to muscle relaxation (25).

In studies on the distribution of EP1 and EP2 receptor in the urothelium and suburothelial layer of the guinea pig bladder, it was found that the EP1 staining was located in urothelial cells and in the suburothelium. Both EP1 and EP2 receptor are expressed by the urothelium and the suburothelial interstitial cells (SU-IC’s) (26). The EP2 expression in the urothelium is clearly stronger than the EP1 expression. Furthermore, unlike in umbrella cells, cyclo-oxygenase 1 (COX 1), was present in basal urothelial cells, making them a possible site of PG synthesis (26). These results suggest that, PG produced by the urothelium could target EP1 and EP2 receptors in the urothelium and suburothelium. Furthermore, these findings indicate that the urothelium of the lateral wall and the lamina propria have a high degree of structural complexity and potential for signaling, despite their relatively low nerve density. Hence it can be suggested that complex signaling systems operate in the urothelium and between the urothelium and lamina propria sub-urothelial interstitial cells (SU-IC’s). 

The basal urothelial cell layer and, to a lesser extent the intermediate layer, express COX I. Hence, this layer is capable of generating PG. The local cellular targets for PG are most probably the intermediate layers and the umbrella cells of the urothelium, and SU-IC’s. Therefore, we can hypothesize that this close spatial arrangement of signaling and responding cells could reflect some functional specialization involving interaction between the basal and outer urothelial cell layers.

It is known that guinea pig SU-IC’s express type 3 muscarinic receptors (27). Since the urothelium may synthesize and release acetylcholine (28), this raises the possibility that SU-IC’s receive multiple inputs like acetylcholine and/or PG. These observations show further, that SU-IC’s also express EP1 and EP2, indicating that they respond to PG. In the intact animal a major source of PGs acting on these cells may be derived from the basal and intermediate urothelial layers. As a consequence, SU-IC’s may be a site of integration of different signals. If some signals such as PG are also under modulation by other urothelial signals (ATP and NO), the physiology of this bladder wall region is particularly complex.

In the muscle layer it has been shown that the EP1 receptor was expressed on smooth muscle cells, on surface muscle interstitial cells (SMIC’s) and on intramuscular interstitial cells (IMIC’s) (29). Muscle staining however, was less intense than stainings on the SMIC’s and IMIC’s. Both SMIC’s and IMIC’s have been suggested as the potential source of PG in the muscle layers before 30 which was confirmed in a recent study (29). This localized expression of the EP1 and EP2 receptors and the sites of PG production (COX 1 expression) indicate an unusual and interesting micro-anatomical arrangement in IC’s.

In addition, the PG production site (COX I) in the IC’s was shown to be different from the site that expresses the receptors, which could imply a classic autocrine arrangement. Moreover, the specialization of discrete signal producing regions and signal responsive regions suggests a further degree of complexity. As EP1 and EP2 receptors act through different mediators in the cell and have been shown to have different and in some cases, opposing effects (30, 31), we make a distinction in our hypothesis between these two receptors. In case of EP1, we propose the hypothesis that the PG system operates to facilitate signal propagation in the IC network. Cell activation would stimulate the COX 1 enzyme and induce local PG production and release, which would then activate an adjacent cell via its EP1 receptor region. The physiological consequence of this structural finding needs further exploration. Based on micro-anatomical evidence however, we can suggest that the network of suburothelial, lamina propria and muscle interstitial cells may form a communicating network (32). Support for this hypothesis can be found in the studies on an isolated lamina propria preparation which have revealed that signals released from urothelium are targeted at the suburothelial cell layer (33).

Similar results as found for EP1 were found in studies on EP2 receptor distribution in the muscle layer. The EP2 receptor was shown to be located on SMIC’s, IMIC’s and to a lesser extent on the smooth muscle cells (30). The EP2 receptor and the COX 1 enzyme were also found to be co-expressed in the same cell. However, a spread and augmentation of a PG signal via EP2 is not likely as in most smooth muscle preparations, EP2 has been shown to cause muscle relaxation (21). In several tissues, EP2 receptors have been shown to increase the formation of cAMP. In the smooth muscle of different organs, this rise in cAMP through EP2 leads then to muscle relaxation (25). It is not yet known whether this is the predominant effect of EP2 in the detrusor muscle.

## 3. The EP1/EP2 Balance Hypothesis

The question to be answered is the possible physiological meaning of the shown EP2 arrangement and it’s relation to the EP1 distribution which was shown earlier. It is known that PGE_2_, may have multiple and sometimes apparently opposing effects on a given target tissue (31, 34). Although no functional experiments have been reported, some possible hypothesis could be put forward. It is explicitly mentioned that these hypotheses are not based on data and need to be confirmed in future functional studies. A possible explanation could be found in the differences in affinity of EP2 and EP1 for PG. It has been shown that the rat EP2 receptor signal transduction pathway is approximately 20-fold more sensitive for PGE_2_ than for EP1, as the concentration of PGE_2_ required to produce a half-maximal response was 37 nM for EP2 against 2.1 nM for EP1 (35). Furthermore, in order to determine the affinity of PGE_2_ for EP1 and EP2, saturation analysis has been done showing KD values of PGE_2_ for EP1 and EP2 be around 24 and 5 nM, respectively (35). Therefore, we hypothesize that under normal conditions; PGE_2_ levels in the detrusor might be such, that the inhibiting EP2 effect predominates the activating EP1 effect, which might result in a stabilization of the muscle layer. Under certain circumstances, the PGE_2_ levels may increase above a certain threshold level in which case the contractile EP1 effect becomes more predominant. Small sub-threshold increases of PGE_2_ might, in such a case, be due to subtle bladder wall irritation, inflammation or stretch. The exact role of the EP2 receptor in the bladder wall remains to be determined by future analysis of the activation, signal propagation and bladder sensory pathways by functional studies. The PGE_2_ signal is suggested to spread out through suburothelial and intramuscular interstitial cells expressing EP1. The narrow balance between the contractile EP1 and the possible relaxant EP2 effect might be a system that modulates the autonomous, non-voiding contractions and relaxation which can become disrupted when PGE_2_ levels reach certain higher levels. Given the fact that there is a cAMP coupling of the EP2 receptor in many other tissues and that the dissociation constant of EP2 is lower than that of EP1, it is suggested that a rise in PG levels may gradually push the balance from a relaxant effect (EP2) towards a contractile effect (EP1). Therefore, PG could have a modulatory role on non-voiding contractions by changing the threshold level for excitability of the network of interstitial cells. This idea is supported by earlier studies showing that frequency and amplitude of the non-voiding activity is reduced by stimulation of an adrenergic/cAMP-linked mechanism (36).

## 4. Autonomous Bladder Activity

Activating IC’s can initiate smooth muscle contraction, possibly via direct electrical coupling through gap junctions (37). This idea was put forward in relation to the coordination of localized contractions in the bladder wall (autonomous activity) as part of a motor/sensory system regulating bladder afferent activity (38, 39). Many studies described and characterized IC’s, which are known to form a network throughout the bladder wall (8, 38, 40, 41). These results show that this network of IC’s extends from the suburothelial layer and runs through the muscle layer and expresses EP1 and EP2 among other receptors. PGE_2_ mediated signals can then be relayed through this network via the PGE_2_ produced by the COX 1 enzyme that was recognized in the basal urothelial cell layer. Studies in the bladder muscle showed that the COX 1 enzyme was also expressed on SMIC’s and IMIC’s. COX 1-positive IC’s were more prevalent in the muscle bundles of the inner muscle than in the outer muscle layers. Signal transduction through coupled IC’s would be more rapid than via the spread of electrical or muscle Ca^2+^ signals. Hence, such coordinated contractions are more likely to be mediated via EP1 and EP2 receptors on IC’s than on muscle cells, since by local PG release, random activation of the latter would result in random uncoordinated activity. Thus, the EP1 and/or EP2 stimulation of muscle IC’s are suggested to be involved in the modulation of the rapid generation of coordinated contractile responses.

## 5. The PGE2 Signal and OAB

The PGE2 signal is suggested to be spread out through sub-urothelial and intramuscular IC’s expressing EP1. The narrow balance between the contractile EP1 and the possible relaxant EP2 effect might be a system that modulates the aforementioned autonomous, non-voiding contractions and relaxation which can become disrupted when PGE2 levels reach certain higher levels. Increased bladder sensation is suggested to be associated with localized contractile activity in the bladder wall of human subjects (micro-motions) which are significantly more prevalent in patients with urgency than in asymptomatic volunteers (42). Hence, urgency is suggested to be associated with autonomous activity of the detrusor and altered micro-motions. If the hypothesis is right and a COX 1–PG–EP1/EP2 system is involved in the modulation of autonomous bladder activity, this system could be a potential therapeutic target. Cyclo-oxygenase inhibiting drugs have been shown to alter normal voiding function in rats as well as bladder hyperactivity induced by chemical irritation (43). Furthermore, it is known that intra-arterial administration of PGE2 to the urinary bladder enhances the micturition reflex (13). It would be interesting to study the possible role of selective drugs which modulate the EP1 and/or EP2 receptor in the bladder. The EP1 receptor is shown to be involved in the initiation of the micturition reflex in normal rats and more specifically, in animals with bladder outlet obstruction (44). Therefore, it is suggested that EP1 contributes to the generation of detrusor overactivity after bladder outlet obstruction (44).

There is hardly any literature about the exact role of the EP2 receptor in the bladder. However, it is known that the normal guinea pig bladder does express EP2 receptors26 and that the combined EP1/EP2 receptor antagonist AH 6809 decreases detrusor contraction in isolated human bladder experiments (45).

## 6. The Effect of COX Inhibition

A link between the PG system and the muscarinic system had been suggested before (32, 33, 46). In the guinea pig bladder, ATP can activate PGE_2_ production by a complex mechanism, involving the purinergic receptors P2X and P2Y (33). This response can be inhibited by indomethacin (a non-selective COX inhibitor) (33). Moreover, clinical data provide another clue for a link between PG and the muscarinic system. It is known that inhibition of cholinergic activity in the bladder by antimuscarinic drugs, does not have a high enough dosage to target the muscle cells but does have an effect in reducing the symptoms of urgency and frequency (47, 48). Therefore, the suggestion is made that anticholinergic drugs could target the mechanisms, operating during the filling phase (49-51) through locally produced substances such as ATP, NO and PG (32, 46). These data show that blocking PG synthesis with indomethacin decreased the cholinergically stimulated autonomous contractions in the isolated bladder. These data suggest that PG could modify normal cholinergically evoked responses.

More in vivo experiments should be performed in order to evaluate the effect of PG on the autonomous activity. A drug combination inhibiting both muscarinic receptors and PG function or production could be proposed as an interesting focus of future research.

## 7. The Role of PDE’s in the Bladder

The cyclic nucleotide mono-phosphates, cyclic AMP and cyclic GMP, are shown to be important intracellular regulators of several physiological processes, including smooth muscle function (52). Possible new target for drug therapy in patients with OAB are the PDE-5 inhibitors. These drugs prescribed to men with erectile disorders, have appeared to exert an inhibitory effect on the symptoms of OAB (53). The role of cyclic nucleotide 5’-cycil (cGMP) in the modulation of detrusor contractile activity, has clearly been shown (54, 55). The intensity and the duration of the existence of intracellular cyclic nucleotides are regulated by PDE’s. Different PDE subtypes have been shown to be present in animal and human bladders, including PDE5 in the rat, PDE1 to PDE5 in the rabbit and PDE1 to PDE5 and PDE7 to PDE9 in the human urinary bladder.

The distribution of the PDE5 enzyme in the urothelium and suburothelium of the guinea pig bladder has been studied in ecperminets where cGMP production was stimulated in fresh guinea pig bladder tissue by an NO-donor, with or without pre-incubation with the PDE5 inhibitor, vardenafil. After that, tissue stainings were conducted with a cGMP antibody. A positive cGMP staining in the tissue indicated the site of action of vardenafil. A few possible sites of action for PDE5 inhibitors in the guinea pig urinary bladder lateral wall can be suggested based on these results, i.e. the urothelium, the endothelium of bladder blood vessels and the network of IC’s. The urothelium can release NO which is needed for cGMP production. PDE5 activity has been shown in the urothelium which suggests that the urothelium can be the site where cGMP is generated.

As both blood vessels and IC’s express the PDE5 enzyme, this signal could be relayed in both ways. Moreover, PDE5 inhibitors are known to have a relaxant effect on the smooth muscle cells of the blood vessel wall, particularly arteries (56) and can therefore increase the blood flow in these arteries (56). This might also be an effect of the cGMP signal generated in the urothelium.

The involvement of nerve fibers is less probable since only a very limited number of nerve fibers were found to stain positive for cGMP after PDE5 inhibition.

As functional studies have not been conducted, only a hypothesis can be forwarded which needs to be further investigated and proven. Based on morphological data it is suggested that PDE5 inhibition has its effect mainly through the nitric oxide mediated cGMP system in the IC’s, rather than having a direct effect on neuronal activity. These data belong to the first set of data available on the site and distribution of PDE5 activity in the bladder and is limited to a study of distribution of the PDE5 activity in the bladder. Therefore, future in vitro and in vivo experiments are needed to reveal the exact physiological meaning of the found PDE5-activity arrangement in the guinea pig urinary bladder.

Although we have reasons to think, that PDE reduces the symptoms of OAB, it is still not known how this effect might be explained. The safety and high tolerability of PDE inhibitors make them an attractive tool, to investigate. Their additional physiological and pharmacological functions, for example, the modulation of intracellular cGMP pools and interactions with other local modulatory mechanisms are interesting for future investigation. Some recent papers show, that PDE inhibition suppressed rhythmic bladder contractions in humans, guinea pig and rat (57). This implies that PDE’s alter micro contractions and exert their effect on OAB via the non-voiding bladder contractions as explained by the autonomous bladder theory. The clinical application of other PDE inhibitors, including those of the PDE4 and recently developed PDE5 inhibitors in storage and voiding disorders, certainly needs more scientific attention and seems likely to become a challenging new treatment alternative in the future. 

## 8. Future Perspectives

A logical next step after the morphological studies would be to study the functional meaning of the structural data found. Although the distribution of EP1 and EP2 in the guinea pig bladder has been studied and presented, in vitro studies using selective EP1 and/or EP2 blockers and agonists are necessary to explore the exact role of these receptors in bladder physiology by assessing excitation–contraction coupling, excitatory synchronization and contractility. One way to do so is in organ bath studies. A further step, and a more challenging one, would be to study the effects of selective EP1 and EP2 blockers and agonist in vivo. Urodynamic measurements of the effects of PG given intravesically in anaesthetized rats have been conducted as a pilot study. A challenging problem is the effect of anesthesia in these animals which could be prevented by using decerebrate animals. The ultimate in vivo study would be to study both voiding parameters as well as urodynamic effects of PG or EP1 and EP2 selective inhibition in an awake animal. These studies require accurate measuring devices. Since the urodynamic measurement devices are wired the moving awake animal will present significant technical challenge. Depending on the results, these studies could lead to phase I and II studies in human subjects. A selective modulation of the EP1 and EP2 receptor could be a therapeutic target in functional bladder disorders such as OAB.

In case of phosphodiesterase inhibitors, future clinical use is closer. As these drugs have been studied and registered for other indications such as erectile dysfunction in men, in vivo studies in human subjects can be conducted on short term. However, from a scientific point of view it is equally important to unravel the exact site of action and role of PDE inhibition with in vitro and in vivo studies as is the case with prostaglandin receptors. This information, in combination with clear and reproducible biomarkers, is needed when we target to modulate different excitatory and or inhibitory control systems at the same time.

In order to achieve this, we need to be able to improve our categorization of patients with symptoms of OAB, and offer more tailored pharmacological treatment. More specifically, patients with OAB but without proven DO on urodynamic studies might need a different therapy than patients that exhibit DO at the same time. A combination of drugs targeting different mechanisms involved in bladder physiology such as PG, cGMP, cAMP, TRPV-channels and muscarinic receptors, could then, due to lower necessary concentrations, provide us with a more favorable effect/side effect balance.
